# Spin State Control of the Perovskite Rh/Co Oxides

**DOI:** 10.3390/ma3020786

**Published:** 2010-01-27

**Authors:** Ichiro Terasaki, Soichiro Shibasaki, Shin Yoshida, Wataru Kobayashi

**Affiliations:** 1 Department of Applied Physics, Waseda University, Tokyo 169-8555, Japan; 2 Waseda Institute for Advanced Study, Waseda University, Tokyo 169-8050, Japan

**Keywords:** thermopower, spin state, oxide thermoelectrics, Heikes formula

## Abstract

We show why and how the spin state of transition-metal ions affects the thermoelectric properties of transition-metal oxides by investigating two perovskite-related oxides. In the A-site ordered cobalt oxide Sr${}_{3}$YCo${}_{4}$O${}_{10.5}$, partial substitution of Ca for Sr acts as chemical pressure, which compresses the unit cell volume to drive the spin state crossover, and concomitantly changes the magnetization and thermopower. In the perovskite rhodium oxide LaRhO${}_{3}$, partial substitution of Sr for La acts as hole-doping, and the resistivity and thermopower decrease systematically with the Sr concentration. The thermopower remains large values at high temperatures (>150 *μ*V/K at 800 K), which makes a remarkable contrast to La${}_{1-x}$Sr${}_{x}$CoO${}_{3}$. We associate this with the stability of the low spin state of the Rh${}^{3+}$ ions.

## 1. Introduction

The spin state is one of the most fundamental concepts in transition-metal compounds/complexes [1]. The Coulomb repulsion from the neighboring oxygen anions changes the *d* energy levels in transition-metal oxides. In a transition-metal ion surrounded with octahedrally-coordinated oxygen anions, the five-fold degenerate *d* orbitals in vacuum are split into the triply degenerate $t_{2g}$ orbitals and the doubly degenerate $e_{g}$ orbitals, and the energy gap between the $t_{2g}$ and $e_{g}$ levels called “ligand field splitting” competes with the Hund coupling. When the ligand field splitting is larger, the *d* electrons first occupy the $t_{2g}$ states to minimize the total spin number. On the other hand, when the Hund coupling is strong, the total spin number is maximized. The former state is called “low spin state”, and the latter “high spin state”. In general, the high spin state is stable at high temperature, because its spin entropy is larger than the entropy of the low spin state.

When the energies of the two spin states are close, various external perturbations such as temperature, pressure and magnetic field can induce the spin state transition/crossover [2]. While the spin state crossover is often observed in transition-metal organic complexes, it is rarely observed in the transition-metal oxides except for cobalt oxides in which the low and high spin states of the Co${}^{3+}$ ion are almost degenerate [3]. *R*CoO${}_{3}$ (*R*; rare-earth) is a prime example in which the magnetization changes dramatically with temperature and physical/chemical pressure [4,5]. A more complicated issue is the possible existence of the intermediate spin state [6], which is still controversial [7,8,9,10].

Since the discovery of the good thermoelectric properties in the layered cobalt oxide Na${}_{x}$CoO${}_{2}$ [11], oxide thermoelectric materials have been extensively investigated [12]. Unlike the state-of-the-art thermoelectric materials, the carriers in the cobalt oxide feel the spin and orbital degrees of freedom that can contribute to the thermopower [13], as was first proposed by Koshibae *et al.* [14]. In this article we report why and how the spin states are related to the thermopower of the perovskite-related oxides by studying two prototypical examples, Sr${}_{3}$YCo${}_{4}$O${}_{10.5}$ and LaRhO${}_{3}$.

## 2. Results and Discussion

### 2.1. Thermopower in correlated systems

First, we briefly review the physical meaning of the thermopower. According to the Boltzmann equation, the electrical current density $\vec{j}$ and the thermal current density $\vec{q}$ are expressed by the linear combination of the electric field $-\vec{\nabla }V$ and the temperature gradient $-\vec{\nabla }T$ as

(1) $$\vec{j}=\sigma \left(-\vec{\nabla }V\right)+\sigma S\left(-\vec{\nabla }T\right)$$

(2) $$\vec{q}=\sigma ST\left(-\vec{\nabla }V\right)+\kappa^{\prime }\left(-\vec{\nabla }T\right)$$

where *σ* is the conductivity, *S* is the thermopower, and $\kappa^{\prime }$ is the thermal conductivity for $-\vec{\nabla }E=0$ [15]. In the absence of temperature gradient $-\vec{\nabla }T=0$, we get

(3) $$\frac{\vec{q}}{T}=S\vec{j}$$

Considering that the left-hand side of this equation is the entropy current density, one can identify the thermopower *S* to the *entropy per charge* when the scattering times involved in $\vec{j}$ and $\vec{q}$ are the same. In this context, the thermopower is a good measure of entropy of carriers, and thus it can detect the entropy due to various degrees of freedom coupled with the carriers.

**Figure 1:**
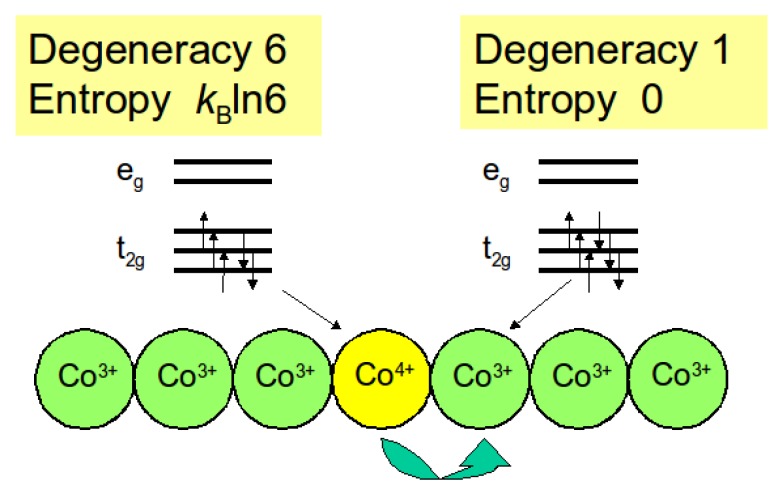
Schematic drawing for the explanation of conduction mechanism of the cobalt oxide proposed by Koshibae *et al*. [14].

Koshibae *et al.* [14] extended the Heikes formula [16] in order to include the spin and orbital degrees of freedom. According to this, the thermopower of the transition metal oxides in the high temperature limit is given by

(4) $$S=\frac{k_{B}}{e}\log\frac{g_{A}}{g_{B}}\frac{x}{1-x}$$

where $g_{A}$ and $g_{B}$ are the degeneracies of the *A* and *B* ions respectively, and *x* is the content of the *A* ions. In the layered cobalt oxide Na${}_{x}$CoO${}_{2}$, the cobalt ions exist as a mixture of Co${}^{3+}$ and Co${}^{4+}$. The magnetic measurement has revealed that they are in the low spin states at 300 K [17]. As schematically drawn in Figure 1, the six electrons in the low-spin Co${}^{3+}$ ion fully occupy the $t_{2g}$ levels, so that it has no other degenerate state. In contrast, in the low-spin Co${}^{4+}$ ion, one electron is removed out of the six electrons, and thus six states are degenerate. Substituting $g_{A}=6$ and $g_{B}=1$ in Equation (4), we evaluate the thermopower to be $k_{B}\log6/e=$150 *μ*V/K. This value is close to the thermopower of Na${}_{x}$CoO${}_{2}$ at 1000 K [18]. This is reasonable, because the Heikes formula is an asymptotic expression of the thermopower in the high temperature limit. Here we ignore the *x* dependent term, because *x* is close to 0.5. This large entropy is evidenced by the specific heat measurement [19], and the thermodynamic properties of Na${}_{x}$CoO${}_{2}$ is compared with those of heavy fermion intermetallics [13]. We should further note that Equation (4) explains why all of the related layered cobalt oxides show large thermopower [20,21,22,23].

This vividly exemplifies the importance of the spin and orbital entropy stored in the transition-metal ions. We emphasize that such entropy is absent in doped semiconductors such as Si, GaAs, and Bi${}_{2}$Te${}_{3}$, which offers a unique design of thermoelectric materials using the transition metal oxides. Previously Kobayashi *et al.* [24] showed by controlling the entropy current that the thermopower can be *negative* for hole-doped semiconducting manganese oxides CaMn${}_{3-x}$Cu${}_{x}$Mn${}_{4}$O${}_{12}$.

### 2.2. The A-site ordered perovskite cobalt oxide

Recently, Kobayashi *et al.* [25] found that Sr${}_{1-x}$Y${}_{x}$CoO${}_{3-y}$ shows a ferromagnetic transition below 340 K for polycrystalline samples in a limited range of the Y content from x=0.20 to 0.25. They found that the ferromagnetism is closely related with the ordering of the A-site cations approximately in a ratio of Sr:Y = 3:1 [26,27,28]. To emphasize this ordering, we will denote this material Sr${}_{3}$YCo${}_{4}$O${}_{10.5}$ (SYCO) in this article. Kobayashi *et al.* [29] further found various similarities to LaCoO${}_{3}$ in the high-temperature transport above $T_{c}$. A significant difference is that the CoO${}_{6}$ volume is larger in SYCO than in LaCoO${}_{3}$, and accordingly the high spin state is stable down to low temperatures. This volume is indeed critical, and the magnetism of SYCO is susceptible against chemical and physical pressure [30]. The magnetization decreases below 190 K for some samples, suggesting that a part of the Co${}^{3+}$ ions go to the low spin state. Kimura *et al.* [31] discovered a metamagnetic transition near 40 T in such samples, and ascribed this to the spin-state crossover induced by an external magnetic field.

**Figure 2:**
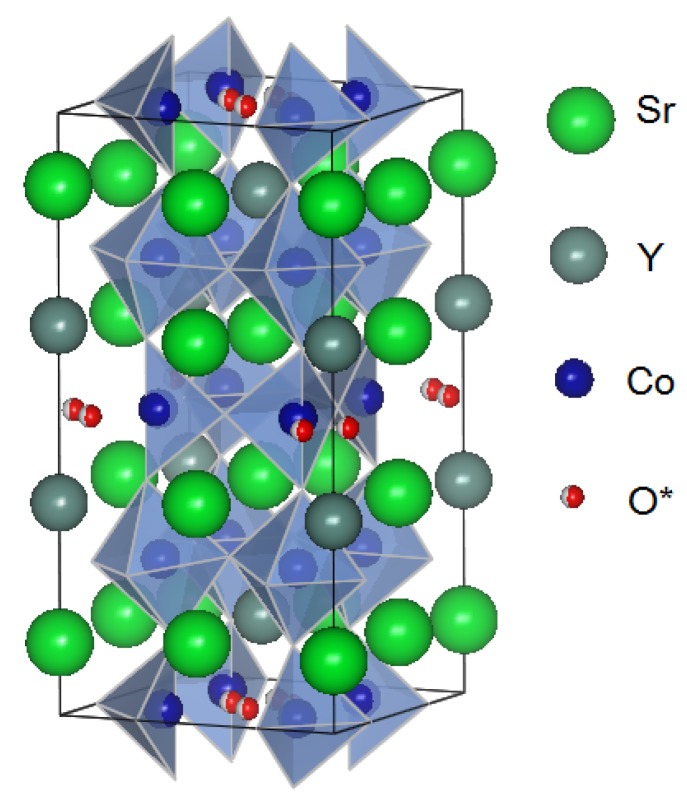
Crystal structure of Sr${}_{3}$YCo${}_{4}$O${}_{10.5}$. O* represents the oxygen site with 25% occupancy. The octahedra and tetrahedra correspond to oxygen networks. The structure is brownmillerite-like, and the octahedra and tetrahedra are alternately stacked along the *c* axis. Sr and Y are ordered along the ab plane, and are stacked along the *c* axis with a periodicity like -Sr-Y-Y-Sr-.

As shown in Figure 2, this particular oxide basically crystallizes in a brownmillerite-like structure, where the octahedral CoO${}_{6}$ layer and the tetrahedral/pyramidal CoO${}_{4.25}$ layer are alternately stacked with insertion of the ordered Sr${}_{0.75}$Y${}_{0.25}$O layer [26,28]. Very recently Sheptyakov *et al.* [32] have shown that the Co ions in the CoO${}_{6}$ layer occupy the intermediate spin state, and those in the CoO${}_{4.25}$ layer do the high spin state. This is, however, an oversimplified picture; Ishiwata *et al.* [33] analyzed the crystal structure of the related oxide Sr${}_{3.1}$Er${}_{0.9}$Co${}_{4}$O${}_{10.5}$ by means of the Rietveld refinement of X-ray diffraction patterns, and found that this oxide exhibits a much more complicated large unit cell with various inequivalent cobalt sites. The same super-structure was observed through the electron microscope by James *et al.* [34].

**Figure 3:**
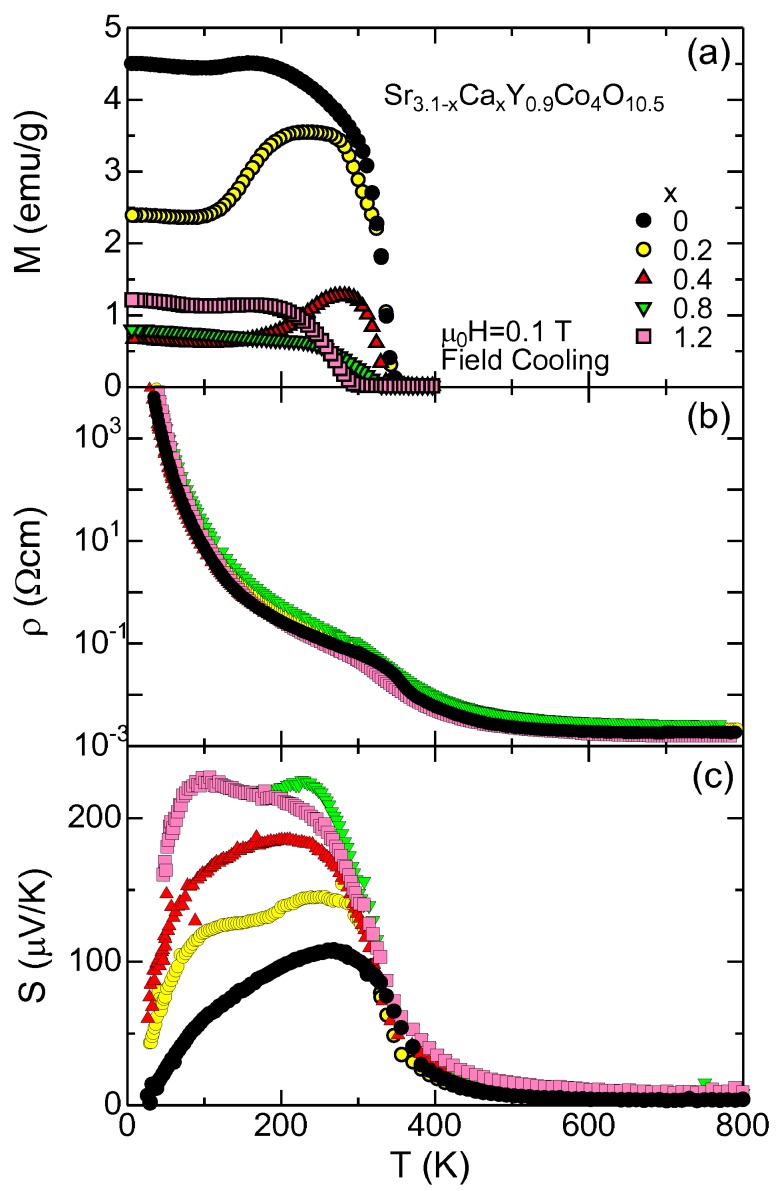
The physical properties of the Ca-substituted SYCO. (a) Magnetization *M* in 0.1 T, (b) resistivity *ρ* and (c) thermopower *S*.

Figure 3 shows the physical properties of the Ca substituted SYCO. In a previous paper [30], we reported the Ca substitution effects for this compounds below 400 K. Since the Sr and Ca ions are divalent, this substitution did not change the oxygen content, but decreased the lattice parameters owing to the smaller ionic radius of Ca${}^{2+}$ ions. As a result, the Ca substitution acts as chemical pressure, which drives the spin state of Co${}^{3+}$ from the high/intermediate spin state of larger volume to the low spin state of smaller volume, as is similar to the case of La${}_{1-x}R_{x}$CoO${}_{3}$ (*R*= Pr [35] and Eu [36]).

Figure 3(a) shows the the magnetization of Sr${}_{3.1-x}$Ca${}_{x}$Y${}_{0.9}$Co${}_{4}$O${}_{10.5}$ in 0.1 T. For x=0, the magnetization rapidly increases below 340 K, indicating the weak ferromagnetism of this compound. With increasing Ca content, the magnetization dramatically drops with a decrease in the transition temperature, which is associated with the spin state crossover driven by chemical pressure. It should be noted that the magnetization of x=0.2 exhibits complicated behavior. It rises below 340 K, takes a broad maximum around 250 K, and goes down below around 200 K. This indicates the competition between the magnetic order and the spin state crossover. The volume of the x=0.2 sample is so critical that the magnetic order becomes unstable below the transition temperature, and some fractions of the Co${}^{3+}$ ions go to the low spin state. For higher substitution, the majority of the Co${}^{3+}$ ions is already in the low spin state at the transition temperature. Owing to unavoidable inhomogeneity due to the solid solution between Sr and Ca, the chemical pressure is somehow inhomogeneous, and a small amount of magnetization survives up to x=1.2.

Figure 3(b) shows the resistivity of Sr${}_{3.1-x}$Ca${}_{x}$Y${}_{0.9}$Co${}_{4}$O${}_{10.5}$. As is clearly seen, the resistivity is almost independent of the Ca substitution. At 800 K, the resistivity is as low as 2-3 mΩcm, where the *d* electrons on the Co${}^{3+}$ ion become itinerant. Kobayashi *et al.* [29] measured the Hall coefficient of x=0 in this temperature range and found that the magnitude is of the order of $10^{-4}$ cm${}^{3}$/C, which corresponds to a carrier density of conventional metals. In this temperature range, all the Co${}^{3+}$ ions are magnetic and metallic, and the decrease of the unit cell volume by the Ca substitution negligibly affects the magnitude of the resistivity. This situation is similar to the high temperature transport in LaCoO${}_{3}$ [37], although the microscopic mechanism is still controversial at present.

The resistivity increases with decreasing temperature, and takes a cusp at the magnetic transition temperature. Since the magnetic Co${}^{3+}$ ions undergo a long range order, they cease to be itinerant, which is detected by an increase of the magnitude of the Hall coefficient [29]. Instead, a small amount of Co${}^{4+}$ ions due to oxygen nonstoichiometry are responsible for electrical conduction. Again, the resistivity is expected to be independent of the Ca content, because the oxygen nonstoichiometry and the content of the Co${}^{4+}$ ions are independent of the Ca content.

In contrast to the resistivity, the thermopower dramatically changes with the Ca content. Figure 3(c) shows the thermopower of Sr${}_{3.1-x}$Ca${}_{x}$Y${}_{0.9}$Co${}_{4}$O${}_{10.5}$. At high temperature around 800 K, the thermopower is of the order of 1 *μ*V/K, which is a typical magnitude for the thermopower of conventional metals. This is consistent with the fact that all the Co${}^{3+}$ ions become itinerant at such temperatures. Toward the transition temperature, the thermopower rapidly increases, suggesting the reduction of the carrier concentration. Below about 300 K, the thermopower exhibits strong Ca dependence. With increasing Ca content, the thermopower largely increases. At 100 K, the thermopower for x=0 is 60 *μ*V/K, whereas that for x=1.2 is 220 *μ*V/K. Since the resistivity is essentially the same value between x=0 and x=1.2, the thermoelectric power factor $S^{2}/\rho$ and perhaps the thermoelectric figure of merit $Z=S^{2}/\rho \kappa$ are enhanced by a factor of (220/60)${}^{2}∼$13. We notice that the resistivity is too high for practical applications, but nevertheless this is a good example that the thermopower can be enhanced with remaining the resistivity unchanged.

This thermopower enhancement is understandable in terms of the spin-state crossover driven by the chemical pressure. In Equation (4), let the A and B ions be Co${}^{4+}$ and Co${}^{3+}$, respectively. Then the degeneracy $g_{B}$ is 1 and 15, respectively, for the low and high spin states of Co${}^{3+}$. Here we used the spin number of S=2 and the orbital number of L=1 for the high spin state of Co${}^{3+}$ [${\left(e_{g}\right)}^{2}{\left(t_{2g}\right)}^{4}$]. We can always assume that Co${}^{4+}$ is in the low spin state ($g_{A}=6$). Given a constant *x*, we thus expect that the thermopower should change by $(k_{B}\ln15)/e=$230 *μ*V/K when the Co${}^{3+}$ ions experience the crossover from the high to low spin state. This value is consistent with the observed value of 220−60=160*μ*V/K, assuming that about 70% of the Co${}^{3+}$ ions go to the low spin state. It should be emphasized that the resistivity is not affected by the spin state crossover of Co${}^{3+}$. The electric charge is carried with the Co${}^{4+}$ ions, where the Co${}^{3+}$ ions work only as the background. On the other hand, when the background has a finite entropy, the back flow of the background entropy influences the thermopower [24,38].

### 2.3. Perovskite rhodium oxide

The perovskite cobalt oxide LaCoO${}_{3}$ has been extensively studied as a possible thermoelectric material [39,40,41,42,43,44]. Androulakis *et al.* [40] reported that slightly doped LaCoO${}_{3}$ is as good as a polycrystalline sample of Na${}_{x}$CoO${}_{2}$ at room temperature. Robert *et al.* [41] extensively investigated the thermoelectric properties of doped *R*CoO${}_{3}$. They found that the ZT values can be improved by properly choosing the rare earth element *R*. Iwasaki *et al.* [42] comprehensively studied the thermoelectric properties of Sr-substituted LaCoO${}_{3}$ from 4 to 1100 K, and found that the thermopower rapidly decreases above 500 K for all samples. One serious drawback of this class of materials is that the Co${}^{3+}$ ions change their spin state from the low spin to the intermediate/high spin state at high temperatures, and become itinerant like conventional metals [3,37]. Owing to this, the thermopower goes down to a small value of the order of 1 *μ*V/K, and decreases ZT at high temperatures. Similar behavior is already seen in SYCO in Figure 3, where the thermopower drops rapidly above around 350 K. Note that the drop occurs at lower temperature than in the case of LaCoO${}_{3}$, because SYCO has a larger unit cell to accept intermediate/high spin state from lower temperatures.

To overcome this drawback, we focus on rhodium oxides. Rhodium is located below cobalt in the periodic table, and thus is expected to have similar chemical properties. In fact, many cobalt oxides have their isomorphic rhodium oxides, and similar transport properties are reported [45,46,47,48,49,50]. An important difference from the Co${}^{3+}$ ions is that the Rh${}^{3+}$ ions are stable in the low spin state at all the temperatures of interest.

**Figure 4:**
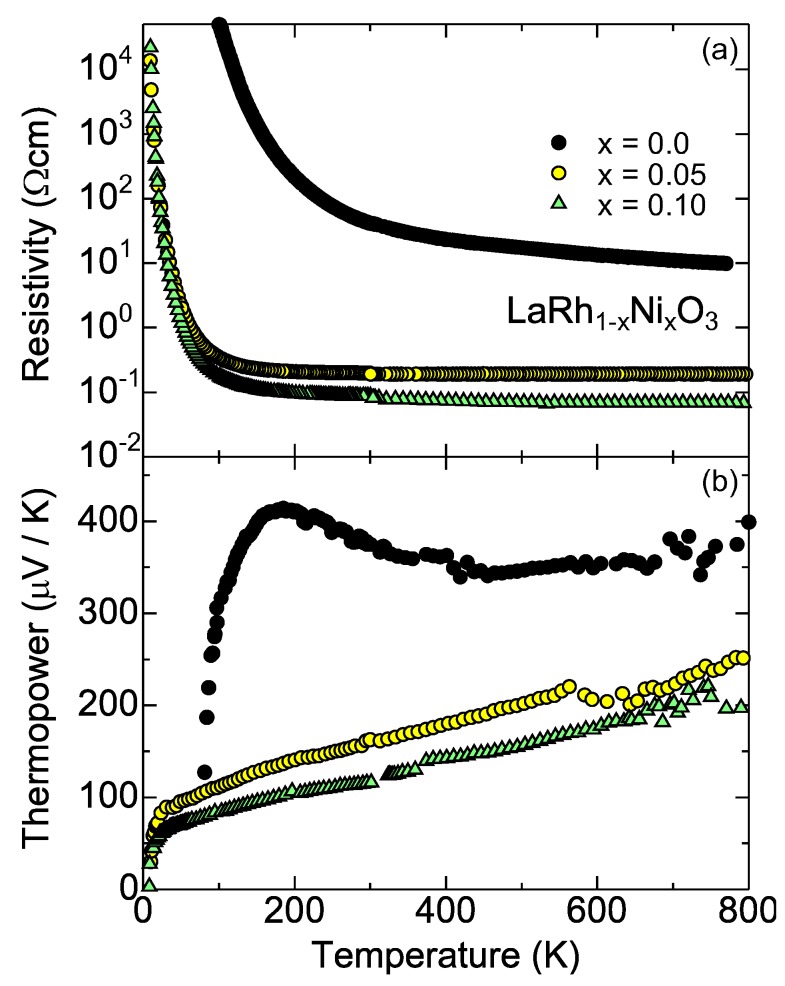
The transport properties of the LaRh${}_{1-x}$Ni${}_{x}$O${}_{3}$ [51]. (a) Resistivity and (b) thermopower.

Shibasaki *et al.* [51] experimentally showed that Ni-substituted LaRhO${}_{3}$ exhibits large thermopower at high temperatures, and has better thermoelectric performance at 800 K. Figure 4 shows the resistivity and thermopower of polycrystalline samples of LaRh${}_{1-x}$Ni${}_{x}$O${}_{3}$. Both quantities systematically change with increasing Ni content, showing that the substituted Ni ion acts as an acceptor. They think that the Ni ions are doped as divalent in the lightly doped region, and induces Rh${}^{4+}$ per Ni${}^{2+}$ to keep the formal valence of the B-site ion to be trivalent (*i.e.*, 2Rh${}^{3+}\to$ Rh${}^{4+}$+Ni${}^{2+}$), They verified this idea by measuring the susceptibility.

One can see that the thermopower remains large values up to 800 K. This makes a remarkable contrast to the thermopower of *R*Co${}_{1-x}$Ni${}_{x}$O${}_{3}$, where the thermopower rapidly decreases at high temperatures owing to the spin state crossover [41]. This large thermopower is consistent with the fact that Rh${}^{3+}$ is stable in the low spin state. In compensation for the large thermopower, the resistivity is higher than that of *R*Co${}_{1-x}$Ni${}_{x}$O${}_{3}$ [41,44]. *d* electrons on the Co${}^{3+}$ sites become itinerant at high temperature, and probably conduct in the wide $e_{g}$ bands [37], whereas the conduction occurs always in the narrow $t_{2g}$ bands in doped LaRhO${}_{3}$. The overlap between the neighboring $t_{2g}$ bands is smaller in the perovskite structure than in the CdI${}_{2}$-type CoO${}_{2}$ block in Na${}_{x}$CoO${}_{2}$.

**Figure 5:**
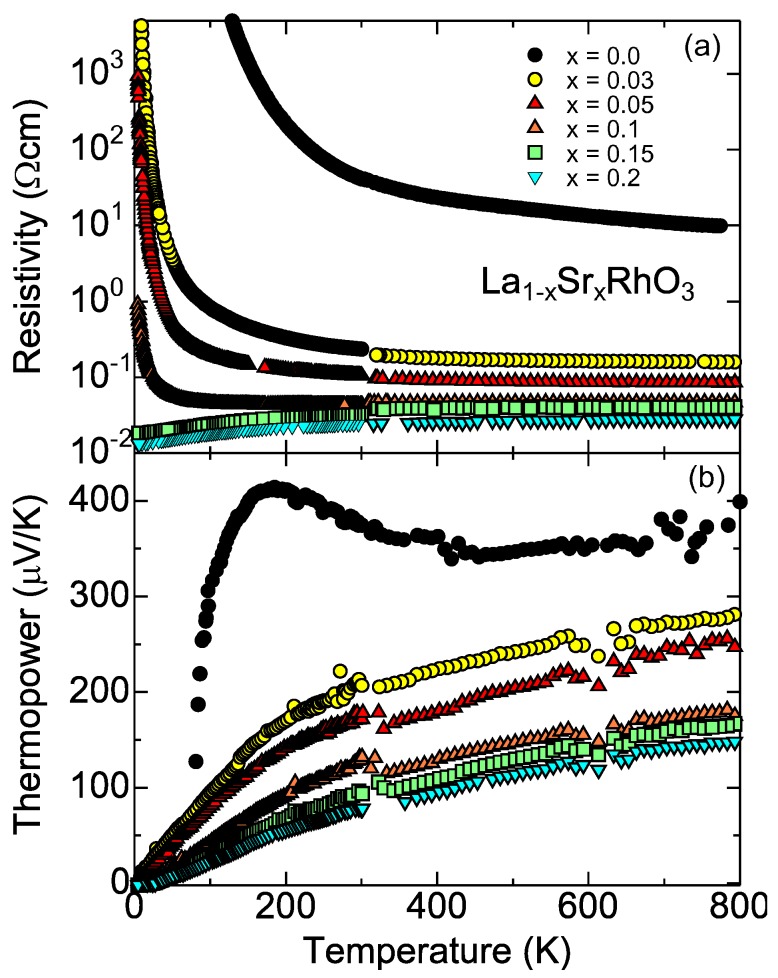
The transport properties of the La${}_{1-x}$Sr${}_{x}$RhO${}_{3}$. (a)Resistivity and (b)thermopower.

Compared with the B site substitution, the A site substitution is easy to control the formal valence of Rh. Figure 5 shows the resistivity and thermopower of polycrystalline samples of La${}_{1-x}$Sr${}_{x}$RhO${}_{3}$. As is similar to Figure 4, both quantities systematically decrease with increasing Sr content, showing that the doped Sr ion acts as an acceptor. The resistivity shows a metal-insulator transition around x=0.15. The critical concentration of x=0.15 is significantly smaller than that of La${}_{1-x}$Sr${}_{x}$CoO${}_{3}$ (∼ 0.3) [52]. In La${}_{1-x}$Sr${}_{x}$RhO${}_{3}$, the electrical conduction occurs only in the $t_{2g}$ bands, and both of Rh${}^{3+}$ and Rh${}^{4+}$ are in the low spin states. In La${}_{1-x}$Sr${}_{x}$CoO${}_{3}$, on the other hand, the doped Co${}^{4+}$ induces the spin state crossover to the neighboring Co${}^{3+}$ to make a spin polaron [53]. The spin polaron can be itinerant at room temperature, where most of the Co${}^{3+}$ ions are magnetic. With decreasing temperature, the low spin state becomes stable for the Co${}^{3+}$ ions away from the Co${}^{4+}$ ions, and as a result, electronic phase separation takes place to localize the spin polaron [54]. As such, the resistivity goes nonmetallic below x=0.3 in La${}_{1-x}$Sr${}_{x}$CoO${}_{3}$ at low temperatures.

The thermopower also make a remarkable contrast to those of La${}_{1-x}$Sr${}_{x}$CoO${}_{3}$ [42]. The thermopower continues to increase up to 800 K for all *x*, and remains larger than 150 *μ*V/K at 800 K, as is already discussed with Equation (4). Although Equation (4) is based on a picture of the localized electrons (strong correlation picture), the temperature dependence seen in Figure 5 is like that of degenerate semiconductors. This suggests that the electronic state is basically understood by a band picture (moderate correlation picture). Actually, when Rh${}^{3+}$ and Rh${}^{4+}$ (Co${}^{3+}$ and Co${}^{4+}$) are both in the low spin states, the electrical conduction may be explained from the band theory because of the absence of local moments. At an early stage of the thermoelectric study in Na${}_{x}$CoO${}_{2}$, Singh [55] already pointed out that the thermopower and specific heat of Na${}_{x}$CoO${}_{2}$ can be quantitatively understood from an LDA calculation. Recently Usui *et al*. [56] have calculated the thermopower of doped LaRhO${}_{3}$ using an ab-initio calculation, which quantitatively agrees with the room-temperature thermopower in Figure 4.

It has been a long-standing problem in conducting transition metal oxides which picture (localized or itinerant electron picture) describes better (e.g., see [57]). Thus it may suffice to say that there exist some anomalous features beyond simple band pictures. One feature is that the thermopower of the Ni-substituted LaRhO${}_{3}$ shown in Figure 4 has a peculiar cusp around 20 K. This is different from the thermopower of conventional metals and semiconductors, and seems difficult to be calculated. Empirically, such temperature dependence is seen in disordered Co/Rh oxides such as B-site substituted LaCoO${}_{3}$ [43], Ca${}_{3}$Co${}_{4}$O${}_{9}$ [20,58], Bi-Sr-Co-O [21,59], and Bi-Sr-Rh-O [45,47]. A second feature is the nontrivial magnetism of the perovskite rhodium oxides; SrRhO${}_{3}$ is an antiferromagnetic metal [60] and La${}_{1-x}$Sr${}_{x}$RhO${}_{3}$ is a Curie-Weiss metal [61]. This indicates that Rh${}^{4+}$ (and possibly a mixture of Rh${}^{3+}$ and Rh${}^{4+}$ as well) is magnetic, which is difficult to predict from the band calculation.

## 3. Experimental Section

Polycrystalline samples were prepared by a solid-state reaction method. For Sr${}_{3}$YCo${}_{4}$O${}_{10.5}$, SrCO${}_{3}$, CaCO${}_{3}$, Y${}_{2}$O${}_{3}$ and Co${}_{3}$O${}_{4}$ were mixed and calcined at 1,100 ${}^{\circ }$C for 12h in air. In order to compensate the evaporation of Co during sintering, we deliberately added an excess 5-mol% Co as starting composition, *i.e.*, the nominal composition was set to be Sr${}_{3.12-x}$Ca${}_{x}$Y${}_{0.88}$Co${}_{4.2}$O${}_{y}$. For further details, see the reference [30]. The calcined product was ground, pressed into a pellet, and sintered at 1,100 ${}^{\circ }$C for 48 h in air. For LaRh${}_{1-x}$Ni${}_{x}$O${}_{3}$ and La${}_{1-x}$Sr${}_{x}$RhO${}_{3}$, stoichiometric amounts of La${}_{2}$O${}_{3}$, SrCO${}_{3}$, Rh${}_{2}$O${}_{3}$ and NiO were mixed, and calcined at 1,000 ${}^{\circ }$C for 24 h in air. The calcined products were thoroughly ground, pelletized and sintered at 1,100–1,200 ${}^{\circ }$C for 48 h in air.

The prepared ceramic samples were characterized by an X-ray diffractometer with a θ−2θ scan mode, and were verified to be in single phase with no detectable impurities. The magnetization-temperature curves were measured using a commercial superconducting quantum interference device magnetometer (Quantum Design MPMS) in a field cooling process of 0.1 T. The resistivity was measured using a four-probe method and the thermopower was measured with a steady-state method with a typical temperature gradient of 1 K/cm. The resistivity and thermopower were measured in a liquid He cryostat below room temperature, and were measured in vacuum on a sapphire substrate painted with RuO${}_{2}$ paste used as a resistive heater above room temperature.

## 4. Summary

In this article, we have shown the thermoelectric properties of the two perovskite-related oxides, and discuss the relationship to the spin states. In the A-site ordered cobalt oxide Sr${}_{3}$YCo${}_{4}$O${}_{10.5}$, partial substitution of Ca for Sr acting as chemical pressure enhances the low-temperature thermopower without increasing resistivity appreciably. This is understood in terms of the spin state crossover driven by the chemical pressure. When the background Co${}^{3+}$ ions go to the low spin state, the entropy flow by the carrier on the Co${}^{4+}$ ion changes, and concomitantly the thermopower changes. In the perovskite rhodium oxide La${}_{1-x}$Sr${}_{x}$RhO${}_{3}$, the thermopower remains large up to high temperatures (>150 *μ*V/K at 800 K), which makes a remarkable contrast to La${}_{1-x}$Sr${}_{x}$CoO${}_{3}$. This is associated with the stability of the low spin state of Rh${}^{3+}$ ions. Through the two examples, we suggest that the spin state control is a unique and effective tool for oxide thermoelectrics.
